# Degradation of methyl orange using hydrodynamic Cavitation, H_2_O_2_, and photo-catalysis with TiO_2_-Coated glass Fibers: Key operating parameters and synergistic effects

**DOI:** 10.1016/j.ultsonch.2024.106772

**Published:** 2024-01-18

**Authors:** Ryma Merdoud, Farid Aoudjit, Lotfi Mouni, Vivek V. Ranade

**Affiliations:** a Laboratoire Matériaux et Développement Durable, Faculté des Sciences et Sciences Appliqués, Université de Bouira, 10000 Bouira, Algeria; b Laboratoire de Gestion et Valorisation des Ressources Naturelles et Assurance Qualité, Faculté SNVST, Université de Bouira, 10000, Algeria; c Department of Chemical Sciences and Bernal Institute, University of Limerick, Ireland

**Keywords:** Degradation rate, External oxidant, TiO_2_-coated glass fibers, Mineralization

## Abstract

Advanced oxidation processes (AOPs) are eco-friendly, and promising technology for treating dye containing wastewater. This study focuses on investigating the removal of methyl orange (MO), an azo dye, from a synthetic wastewater through the use of hydrodynamic cavitation (HC), both independently and in combination with hydrogen peroxide (H_2_O_2_), as an external oxidant, as well as photocatalysis (PC) employing catalyst coated on glass fibers tissue (GFT). The examination of various operating parameters, including the pressure drop and the concentration of H_2_O_2_, was systematically conducted to optimize the degradation of MO. A per-pass degradation model was used to interpret and describe the experimental data. The data revealed that exclusive employment of HC using a vortex-based cavitation device at 1.5 bar pressure drop, resulted in a degradation exceeding 96 % after 100 passes, equivalent to 230 min of treatment (cavitation yield of 3.6 mg/kJ for HC), with a COD mineralization surpassing 12 %. The presence of a small amount of H_2_O_2_ (0.01 %) significantly reduced the degradation time from 230 min to 36 min (16 passes), achieving a degradation of 99.8 % (cavitation yield of 6.77 mg/kJ for HC) with COD mineralization rate twice as much as HC alone, indicating a synergistic effect of 4.8. The degradation time was further reduced to 21 min by combining HC with PC using TiO_2_-coated glass fibers and H_2_O_2_, (cavitation yield of 11.83 mg/kJ for HC), resulting in an impressive synergistic effect of 9.2 and COD mineralization twice as high as the HC/H_2_O_2_ system. The results demonstrate that HC based hybrid AOPs can be very effective for treating and mineralizing azo dyes in water.

## Highlights

• Evaluated effectiveness of advanced oxidation processes for degradation of methyl orange.
• Hydrodynamic cavitation (HC) was found to enhance effectiveness of H_2_O_2_ and photocatalysis.
• The HC with H_2_O_2_ and photocatalysis with TiO_2_ coated glass fibers was found to be best.
• The maximum synergy factor for this combination of three AOPs was found to be 9.2.

## Nomenclature

β: Ratio of flow rate through the cavitation device and working volume (Q/V, s^−1^).
$C_{in}$: Inlet concentration of MO (ppm)
C: Concentration of MO in the holding tank (ppm)
Ca: cavitation number (-)
${COD}_{0}$: Chemical oxygen demand at t = 0
${COD}_{t}$: Chemical oxygen demand at t(min)
$d_{T}$: Characteristic diameter of the vortex based-HC device (m)
ΔP: Pressure drop across cavitation device (bar)
Eu: Euler number (–)
Φ: per pass degradation factor (-)
k: First-order rate constant (min^−1^)
n: Number of passes through cavitation device (–)
Q: Recirculating flow rate through cavitation device (LPM)
Ρ: Liquid density (kg/m^3^)
$P_{2}$: Pressure at the outlet (kPa)
$P_{v}$: vapour pressure (kPa)
Re: Reynolds number (-)
t: Treatment time (min)
v: Throat velocity for vortex-based cavitation device (m/s)
V: Volume of the solution in all the setup (including the holding tank, the pump and the pipes) (L)
$Y_{\mathrm{cav},HC}$: The cavitation yield (mg/kJ)

## 1 Introduction

One of the major contributors to water contamination are the textile industries, because of their large quantities of dye containing wastewater; a problem that needs to be remedied on a global scale. On an annual basis, ∼ 10^8^ tons of dyes are manufactured, and a significant fraction of it is used by the textile sector around the world [1]**.** This extensive utilization gives rise to a substantial volume of wastewater, laden with persistent organic pollutants (POPs), primarily comprising azo dyes [2]. The environmental repercussions of such wastewater are profound, as it poses a considerable threat to ecosystems [3] and human health [4], attributed to the high toxicity, mutagenicity, and carcinogenicity [5].

Several conventional wastewater treatment techniques, such as adsorption [6], biological treatment [7], coagulation [8], membrane filtration [9] etc., involve the transfer of the pollutant from one medium to another without destroying it, in addition to the high operational cost and time-consuming, with low efficiency. Recognizing these shortcomings, there is a compelling need to explore advanced and eco-friendly alternatives to treat dye-containing wastewater, and herein lies the significance of advanced oxidation processes (AOPs). AOPs such as Fenton process [10], ozonation [11]**,** hydrogen peroxide [12], photocatalysis [13], and others, are capable to degrade and mineralize complex organic pollutants in aqueous solutions, by the generation of powerful non-selective radicals like OH**^.^** [14]. These AOPs are eco-friendly since they focus on complete mineralization of pollutants with high efficiencies.

In recent years, one of the promising AOPs for the treatment of wastewater is hydrodynamic cavitation (HC) [15], [16], [17]. HC is a process of formation, growth, and collapse of gas/vapor filled cavities (microbubbles). These cavities are generated by realizing a low-pressure region (close to vapor pressure of water at operating temperature) within a cavitation device. Turbulence fluctuations in such a low-pressure region generate cavities. When such cavities travel to high pressure regions and are exposed to turbulence pressure fluctuations, they collapse and under certain circumstances lead to local hot spots and highly oxidizing radicals [18]. HC has proven its effectiveness in the elimination of POPs present in synthetic and real wastewater (industrial scale) [19] sourced from diverse sectors including the agricultural [20], packaged food [21], textile [22], and pharmaceutical [23].

While numerous studies have investigated the application of HC for treating dye-containing wastewater, the standalone use of HC has shown limitations in achieving high degradation extents [24], [25], [26]. Several efforts are therefore being made towards combining HC with other AOPs [27], [28], [29]. Recent studies have demonstrated a synergistic effect of combining HC with photocatalysis (PC) [30], [31], and HC/H_2_O_2_ has also shown an appealing approach, for dye degradation [32]. Key studies on use of HC coupled with different AOPs to accelerate the process of pollutant degradation are summarized in Table 1.

**Table 1:** A summary of key studies on hybrid advanced oxidation techniques.

Oxidation processes used	Pollutant (s)	Results	Reference
HC (Venturi), zero-valent iron (ZVI), and sulfite	Direct Red 83 (DR83)	95.54 % of dye degradation using HC/ZVI/sulfite process more effective than the individual processes (<6% for ZVI and sulfite, and 68.21 % for HC)	[33]
HC (orifice), potassium persulfate (KPS), and hydrogen peroxide (H_2_O_2_)	Ponceau 4R, Tartrazine, and Coomassie Brilliant Blue (CBB)	92.27 %, 50.1 %, and 42.3 % were obtained by applying the combined process for CBB, Tartrazine, and Ponceau 4R, respectively.	[34]
		A synergetic coefficient of 2.51 was obtained by HC-KPS-H_2_O_2_ proving the effectiveness of this combined process. The combined HC-KPS-H_2_O_2_ procedure outperformed the sole HC treatment method by 98 %, based on an analysis of cavitation yield efficiency	
HC (orifice), Na2S2O8 and O3	Methylene blue	The combination of HC with ozone is better than that of adding Na_2_S_2_O_8_ with MB degradation rate of over 99 %, and a synergy index of 3.58	[35]
HC (Orifice), H_2_O_2_, Fenton, photo-Fenton, photolytic (UV irradiation), and photocatalytic process (UV irradiation + TiO2)	Methylene blue, Methyl orange, and Rhodamine-B	HC/H_2_O_2_ showed 100 % decolorization over 40 min for a molar ratio of 1:40 (ternary dye: H_2_O_2_) with a higher synergetic effect of 28.97, whereas, HC/Fenton and HC/photo-Fenton process showed 98 % decolorization with synergistic effect of 6.285 and 4.923 respectively at a dosage of 1:30 M ratio of FeSO4:H_2_O_2_. HC/Photolytic process showed 74.53 % decolorization with a synergistic effect of 1.801, and HC/PC showed 82.13 % decolorization with a synergistic effect of 2.11 in 120 min of treatment at optimum conditions	[36]
HC (venturi), Fe3 + -doped TiO2	Rhodamine B	The best degradation was obtained (91.11 %) using HC combined with Fe3 + -doped TiO2 with 0.05:1.00 M ratio of Fe and Ti	[27]
HC (venturi), NaCl, H_2_O_2_ and NaCl, NH4Cl, and Na2SO4	Methyl orange (MO)	A degradation of 30 % using HC alone, 70 % using HC/H_2_O_2_, as well as HC/NaCl, however the combined NaCl and H_2_O_2_ had a negative effect. HC/ NaCl, NH4Cl, and Na2SO4 improved the dye decolorization to 90 %	[37]

The main goal of this work is to investigate the degradation and the mineralization of methyl orange (MO), a hazardous azo-dye model pollutant widely used in the textile industry [38]. We present here results of only HC, HC with H_2_O_2_, as well as HC (and HC + H_2_O_2_) coupled with PC. This study integrates a vortex-based hydrodynamic cavitation with a novel immobilized photocatalyst: TiO_2_-coated glass fibers (GFT). Vortex-based cavitation is known for its superiority over traditional orifice or venturi-based methods as highlighted by Ranade and co-workers [39], [40], [41]. This device offers early inception and notably lower energy consumption compared to conventional alternatives. Previous studies comparing the performance of these devices consistently reveal a significantly better cavitation yield, measured in milligrams of pollutant degraded per unit of energy, for vortex-based cavitation devices [42]. Notably, this work represents the first report showcasing the synergy among vortex-based hydrodynamic cavitation, hydrogen peroxide, and the fiber-coated photocatalyst. The use of the TiO_2_-coated GFT not only overcomes limitations associated with conventional cavitation but also presents a unique solution to suspended TiO_2_ powder challenges such as agglomeration which typically requires post-treatment steps like filtration [43]. The per-pass degradation model was used to describe the experimental data of MO degradation, in batch systems, and interpret the performance of HC alone and combined with H_2_O_2_ and PC. Effectiveness of the treatment was assessed by calculating cavitation yield in terms of mass of dye degraded per unit energy consumption. The presented results offer a useful basis for developing a large-scale treatment of dye wastewater.

## 2 Material and methods

### 2.1 Materials

Methyl orange (molecular weight: 327.334 g mol^−1^; molecular formula: C_14_H_14_N_3_NaO_3_S) was purchased from Thermo Fisher Scientific. For each experiment, distilled water was used to prepare the synthetic MO solutions. Sulphuric acid (Sigma Aldrich, 98 %) was used for adjusting the pH of the solution. Hydrogen peroxide (Thermo Fisher Scientific, 30 %v/v),) was used to test its improvement to the process oxidant capacity. The TiO2-coated glass fiber tissue (GFT) was used as a photocatalyst supplied by Ahlstrom Research and Services [44]. All purchased chemicals were applied as received.

### 2.2 Experimental setup

**Fig. 1:**
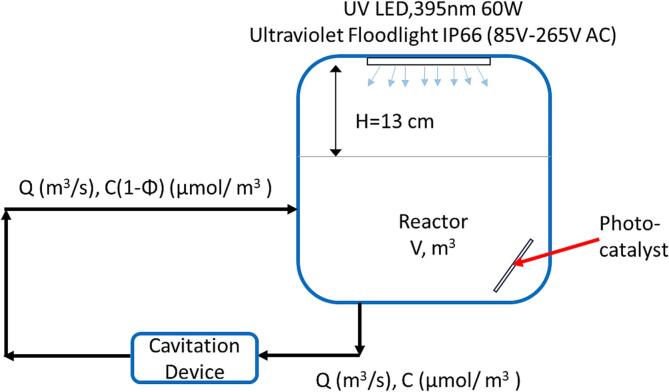
Schematic of the experimental set-up.

Degradation experiments were performed in a lab-scale experimental setup represented schematically in Fig.1. The set-up was designed in such a way to enable us to investigate hydrodynamic cavitation and photocatalysis independently as well as in a coupled mode. The photographs of the set-ups are depicted in the supplementary information (SI) as Fig. S1 and Fig. S2 respectively. The hybrid system is composed of a holding tank, with a maximum capacity of 2.5 L, from which the liquid is pumped using a centrifugal pump with an electric power of 0.75 kW, and integrated manual pressure drop control, which ensures liquid circulation through the pipe to the vortex-based cavitation device (with throat diameter, d = 3 mm). The cavitation device was made of aluminum and was based on the design disclosed by Ranade et al. [40]. The temperature of the holding tank was not controlled but was monitored during the experiment. A 60 W 395 nm UV light LED was mounted horizontally at the top of the aluminum photoreactor with a volume of 43.952 cm^3^ (41H × 32L × 33.5 W cm), 13 cm from the surface of the liquid, as a light source for photocatalytic processes. The TiO_2_-coated glass fiber tissue (GFT) has been diagonally immersed in the solution. Throughout the experiments, the solution was stirred at 200 rpm. The system's liquid flow rate was determined at different pressure drops by measuring 1L at a specific time. The characteristic pressure drop versus flow rate curve of the vortex-based cavitation device is presented in Fig. S3.

### 2.3 Experimental methodology

All experiments were carried out in 2.5 L of methyl orange aqueous solution of a concentration range of 10 to 20 ppm and a pH adjusted to a value close to 2.0 according to previous works [37], [45]. The preliminary experiments have indicated that vortex-based hydrodynamic cavitation efficiency is independent of initial concentration as reported by Ranade et al. [15]. To minimize the usage of chemicals while ensuring the accuracy of analytical techniques, the study opted for the initial dye concentration of ∼ 10 ppm that still allowed for precise measurements. The temperature varied between 30 °C and 47 °C. Three series of experiments were conducted in our work. In the first series, dye degradation was carried out using only hydrodynamic cavitation at different inlet pressures ranging from 1.5 to 3.5 bar for a number of passes equal to 100, in order to determine the ideal inlet pressure for the maximum degradation. The outlet is exposed to atmospheric pressure. Therefore, inlet pressure (stated as gauge pressure) is same as the pressure drop across the cavitation device. The experimental conditions used in the HC experiments are listed in Table 2. The calculation of cavitation number for the vortex-based cavitation devices is not straightforward. Ranade et al. [39] have suggested that cavitation number for vortex-based cavitation devices may be calculated based on maximum tangential velocity in the vortex chamber. Recently Gode et al. [46] proposed a correlation of pressure drop across the cavitation device and maximum tangential velocity in the vortex chamber. Based on that, the values of cavitation numbers were calculated as:

(1) $$\mathrm{Ca}=2\left(\frac{P_{2}-P_{v}}{\Delta P}\right)=\;\frac{2}{\Delta Pinbar}$$

**Table 2:** Experimental parameters used for HC experiments.

ΔP(bar)	1.5	2	2.5	3	3.5
Q(L/min)	1.1	1.3	1.5	1.7	1.9
$V_{T}$(m/s)	2.6	3.1	3.5	4.0	4.5
Re**[**$\left(\frac{d_{T}V_{T}\rho }{\mu }\right)]$	7785	9200	10.616	12.031	13.447
Ca**[**$\;2\left(\frac{P_{2}-P_{v}}{\Delta P}\right)]$	1.33	1	0.8	0.67	0.57

$d_{T}$ is characteristic diameter, $V_{T}$ is velocity based on characteristic diameter, ΔP is pressure drop across the HC device, Q is flow rate through HC device, Re is Reynolds number and Ca is cavitation number, $P_{2}$ is pressure at the outlet and $P_{v}$ is vapour pressure.

In the second series, the synergistic effect of HC mixed with different concentrations of H_2_O_2_ (0 % v/v, 0.001 %v/v, 0.005 %v/v, 0.01 %v/v, 0.1 %v/v, and 1 %v/v) was studied in terms of dye degradation, over 60 min, using the optimum inlet pressure established in the first series of experiments. Finally, MO degradation was investigated using a hybrid system consisting of HC and PC at optimum inlet pressure and H_2_O_2_ parameters, during 60 min of treatment using TiO_2_-coated GFT (5 cm × 5 cm) (Fig. S4), knowing that the MO solutions were placed in the dark, stirred at 200 rpm, for 60 min to reach the adsorption and desorption equilibrium before switching on the lump and starting the photocatalysis process [47]. The TiO2-coated GFT catalyst was characterized using X-ray diffraction (XRD) patterns (/spectra) obtained on an Empyrean X-ray diffractometer using Cu-K⍺ irradiation at a scan rate of 0.1^◦^ 2θ S^−1^. The accelerating voltage and the applied current were 45 kV and 40 mA, respectively. The scanning electron micrographs (SEM) were obtained on an SU70 Hitachi microscope at a voltage of 5 KV after coating the sample with gold for 1 min at 20 mA. The sample of these characteristics are included in Section S1 of the supplementary information. All dye degradation experiments were performed in triplicate. The errors were found to be less than 5 %.

### 2.4 Analysis and processing of data

The collected samples were analyzed using a UV–Vis spectrophotometer (Shimadzu UV1800) (Fig. S5), to follow the variation in absorbance as a function of time at a specific wavelength depending on the pH of the medium. The intensity of the peak corresponding to a maximum absorbance of the MO solution at pH = 2 was observed at 507 nm. The concentrations of MO solutions were indirectly monitored using the calibration curve (Fig. S6). All the concentration values estimated through UV measurements were considered real or accurate concentrations and were used for all subsequent calculations.

It is known that pseudo-first-order kinetics have been employed in many articles providing experimental findings on pollutant degradation via hydrodynamic cavitation [32], [33], [34], [35], [36], [37], [38]. However, Ranade et al. [15] have shown that this approach suffers from some disadvantages. For example, the pseudo-first order kinetics approach, under the same operating conditions, could forecast two different values of the effective rate constant for identical hydrodynamic cavitation reactors, but with different containment volumes. In light of this, we have used the per-pass degradation factor (Φ), which is dependent on the reactor design and operating conditions but not on volume of the holding tank used in the experiments. The approach is particularly useful to comprehend and interpret how pollutants degrade and optimize key operating parameters for the best HC effect [48]. However, for comparing the performance of the HC system with other hybrid options and for quantifying synergistic effects, we have used the pseudo kinetics approach following the accepted trend in most of the published studies using hybrid AOPs.

The overall behavior of a typical cavitation-based water-treatment setup shown in Fig.1. can be modeled as [49]:

(2) $$V\frac{dC}{dt}=Q\Phi C\;\;\;or\;\;\;V\frac{dC}{dt}=-k_{\mathrm{eff}}C$$

where C is a concentration of pollutants, V is a working volume (holding tank volume and volume of piping including the pump), Q is a flow rate through cavitation device, $k_{\mathrm{eff}}$ is an apparent degradation rate constant, and Φ is a per-pass degradation factor. In principle, it is possible to account for the influence of temperature by considering an activation energy. However, in this work, we have not considered the activation energy and have reported the effective value of per-pass factor, Φ or effective rate constant, $k_{\mathrm{eff}}$ estimated over the range of temperature used in the experiments. The number of passes through the cavitation device are related to the residence time of the holding tank (which is a ratio of the holding tank volume and the flow rate through the cavitation device, V/Q, s) and operating time. This relationship may be written as:

(3) n=βt

where n is the number of passes, and β is an inverse of the residence time (Q/V, s^−1^). Chemical oxygen demand (COD) of the MO solutions, was measured by using DR 1900 spectrophotometer (Hach) for all samples analysis, after digesting 2 mL of the sample to the commercial cuvette tests COD reagent (LCI400, 0–––1000 mg/L) for 120 min at 148 °C using LT 200 digester (Hach-Lange, Germany) (Fig. S7**, and** Fig. S8 respectively). The %COD destroyed was calculated as:

(4) $$\%COD=\frac{{\mathrm{COD}}_{0}\;-\;{\mathrm{COD}}_{t}\;}{{\mathrm{COD}}_{0}}\times 100$$

where ${\mathrm{COD}}_{0}$ and ${\mathrm{COD}}_{t}$ are the values of COD corresponding to the MO solutions before and after treatment respectively.

Hydrodynamic cavitation involves fewer operational complexities compared to PC or the addition of chemicals like H_2_O_2_. Therefore, to enhance the precision of system efficiency assessment, we propose concentrating on the cavitation yield of HC ${(Y}_{\mathrm{cav},HC}$), providing a practical and cost-effective metric for efficiency evaluation. The cavitation yield ${(Y}_{\mathrm{cav},HC})$ is a critical parameter in evaluating the efficiency of the hydrodynamic cavitation (HC) process. It is expressed as the mass of pollutants degraded per unit of energy consumption and is calculated as:

(5) $$Y_{\mathrm{cav},HC}=\frac{V\;\left(C_{\mathrm{in}}-C\right)}{\Delta PQt}$$

where $C_{\mathrm{in}}$ is initial dye concentration and C is dye concentration at time t. $Y_{\mathrm{cav},HC}$ is reported in the units of mg of dye degraded/kJ of energy spent on HC.

## 3 Results and discussion

Initially, results of experiments using only HC for dye degradation are discussed. Influence of adding H_2_O_2_ along with the HC process on the MO degradation was then discussed for optimal condition identified from only HC experiments. Existence of synergy between externally added H_2_O_2_ and HC was evaluated. Before carrying out the photocatalysis experiments, the physical properties of the catalyst used were characterized. We used diffraction analysis, a non-destructive technique, to determine the crystalline structure of TiO_2_-coated GFT **(**Fig. S9**).** This method enabled us to define the crystallite size of the TiO_2_ nanoparticles, which has a significant effect on the material's electrical and optical properties. The SEM was used to obtain information about the sample's surface topography and composition **(**Fig. S10**)**. After characterizing the photocatalyst, the possibility of using the hybrid system, HC/H_2_O_2_/PC, at optimum operating conditions of combination of HC and H_2_O_2_, was investigated in terms of MO degradation. Extent of mineralization was also investigated. The results are discussed in the following sub-sections.

### 3.1 Application on only hydrodynamic cavitation for degradation of MO

The flow rate through the HC device or in other words, pressure drop across the HC device is one of the key parameters controlling the effectiveness of hydrodynamic cavitation in the degradation of organic pollutants [50]. Ranade et al. [39] have reported that the vortex-based cavitation device exhibits inception at a pressure drop of around 0.8–1 bar. However, the actual inception point may vary to some extent from the stated value, depending on operating temperature, concentration of dissolved gases, presence of impurities, etc. In order to avoid any ambiguity inherent in the starting point, and to ensure reproducibility and control the size of error bars on the data to be reported, the influence of inlet pressure or pressure drop across the cavitation device was studied over the range of pressure drop of 1.5 and 3.5 bar. The pH was kept constant at 2 for all these experiments. The samples were collected after 0, 10, 30, 60, 80, and 100 passes and were analyzed for MO concentration. Hydrodynamic cavitation leads to the formation of cavities and their collapse in the cavitation device, which generate very high local T and P (P = 1000 atm, T = 10000 K) which generates strongly oxidizing radicals responsible for the removal of pollutants present in the solution. Sarvothaman et al. [51] have simplified the pollutant degradation reactions as:

Cavity collapse → OH**^.^** and other radicals (R**^.^)**(6)

R**^.^** + pollutant → intermediates → CO2 + H_2_O(7)

**Fig. 2:**
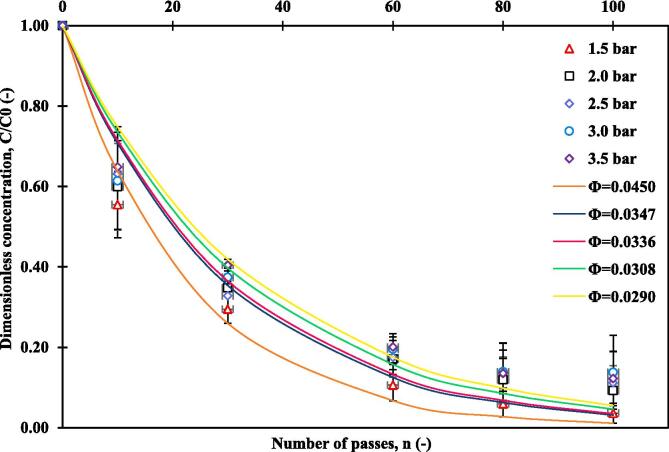
Degradation of methyl orange versus number of passes for various levels of inlet pressure; V = 2.5 L, pH = 2, and initial concentration of 10 ppm.

As the dye containing water is circulated through HC device, every pass through device allows exposes dye molecules to collapsing cavities which leads to degradation of dye molecules via reaction represented by Equation (7). The observed reduction in MO concentration with respect to number of passes for different values of pressure drop are shown in Fig. 2.

It can be seen that the highest degradation of 96.4 % was obtained at inlet pressure of 1.5 bar. Beyond 1.5 bar pressure, the extent of degradation was found to decrease significantly. Considering that the power consumption per unit weight of treated wastewater is proportional to pressure drop, the lowest pressure drop considered in this work, 1.5 bar which also exhibited maximum degradation can be considered as an optimum pressure drop. It should be noted that the overall effectiveness of degradation of pollutant via hydrodynamic cavitation depends on product of two factors: number density of collapsing cavities (number/m3) and intensity of cavity collapse. As pressure drop across the cavitation device increases, driving force for cavitation increases and, consequently, number density of cavities increase. However, as more and more cavities co-exist in liquid, effective compressibility of the medium increases. This leads to reduction in intensity of cavity collapse, and hence a reduction in the number of hydroxyl radicals generated, resulting in a lower rate of pollutant degradation [39]. The extent of degradation for the operating pressure drops of 2 to 3.5 bar was within 10 %.

Following the works of Ranade et al. [48], [49], the per-pass degradation model was used to describe the batch experimental data. It can be seen from Fig. 2 that the per-pass degradation factor, which depends on the generation of hydroxyl radicals as mentioned above, reduces with increase in pressure drop and is in the range of 0.029 to 0.045. The pressure drop of 1.5 bar exhibits highest degradation with per-pass degradation factor of 0.045. This was selected for subsequent experiments.

### 3.2 HC combined with H_2_O_2_: Synergistic effect

**Fig. 3:**
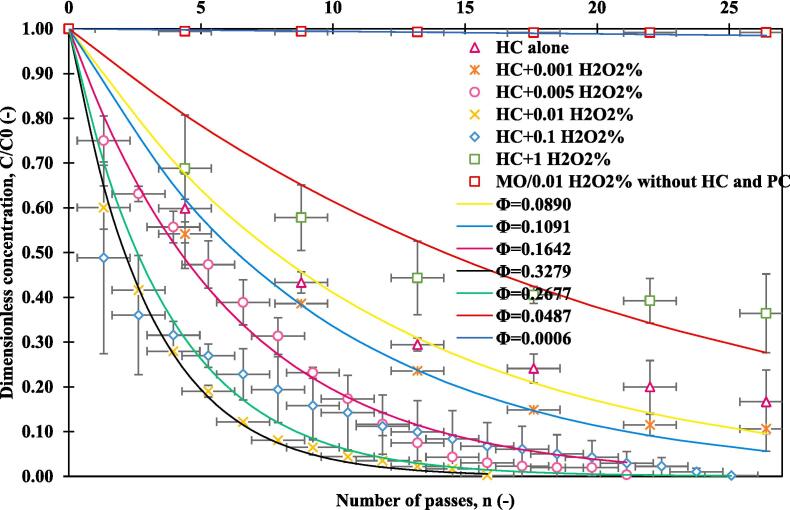
Degradation of methyl orange versus number of passes for different concentrations of hydrogen peroxide; V = 2.5 L, pH = 2 and MO initial concentration = 10 ppm, ΔP = 1.5 bar.

Among the potent oxidizing agents, hydrogen peroxide is widely used in oxidation reactions as well as recently, to intensify cavitation processes, in terms of removing persisting organic pollutants [23], [52], [53]. Added H_2_O_2_ with hydrodynamic cavitation is expected to increase the generation of free radicals and therefore enhance the extent of degradation of pollutants [32], [54]. Fig. 3 shows the results of a series of experiments using HC alone, a blank MO test with H_2_O_2_ in the absence of HC, and different H_2_O_2_ concentrations combined with HC, as a function of number of passes in the optimum pressure drop of 1.5 bar. The maximum degradation values, using HC alone, were 83.3 %, and for the blank MO/H_2_O_2_ alone was 0.9 %, achieved after 26 passes equivalent to 60 min of treatment. The combination of H_2_O_2_ with hydrodynamic cavitation improved overall degradation of MO from 83.3 % to 99.8 % by using the concentration of H_2_O_2_ up to 0.1 %. It was observed that initially, increase in H_2_O_2_ concentration lead to increase in the rate and extent of degradation. Addition of 0.001 %v/v H_2_O_2_ lead to increase in degradation from 83.3 % (only HC) to 89.4 % in 26 passed. Increase in H_2_O_2_ concentration to 0.005 % led to MO degradation of 99.6 % in 21 passes and to 0.01 % H_2_O_2_ concentration led to MO degradation of 99.8 % in 16 passed. Further increase in H_2_O_2_ concentration to 0.1 % however did not increase the extent of degradation (and in fact slightly reduce the rate – number of passes increased from 16 to 25 for 99.8 % degradation).

High H_2_O_2_ concentration like 1 % v/v H_2_O_2_ may cause enhancing scavenging of radicals due to recombination (self-scavenging of radical species), as well as the negative effect of the oxidant which in turn reacts with these OH radicals formed [55]. These potential recombination reactions may be represented as [32]:

OḢ+ OḢ → H_2_O_2_(8)

OḢ+ H_2_O_2_ → HO_2_ ˙ + H_2_O(9)

OḢ+ HO_2_**˙** → H_2_O + O_2_(10)

It can be seen from Fig. 5 that the per-pass degradation factor increases with H_2_O_2_ concentration, reaching a maximum of 0.328 at an optimum of 0.01 %v/v H_2_O_2_, then decreased sharply to 0.0487 at 1 %v/v H_2_O_2_. The experimental results at different concentrations indicate that the external addition of 0.01 %v/v of H_2_O_2_ was the best among the considered cases which led to 99.8 % degradation of MO with just 16 passes (equivalent to 36 min of treatment time).

The results of MO degradation in terms of time of treatment, % degradation and calculated effective rate constants $k_{\mathrm{eff}}$, per pass degradation factor Φ, synergistic coefficients, and cavitation yield $Y_{\mathrm{cav},HC}$ for this second series of experiments are listed in Table 3. The first order (-ln(C/C0) vs time) kinetics effective rate constants range from 0.0385 min^−1^ and 0.0161 min^−1^ corresponding to 0.001 %v/v and 1 %v/v H_2_O_2_ respectively. The synergy between HC and H_2_O_2_ was also established using the rate constants obtained for the degradation of MO using HC and H_2_O_2_ separately. The synergistic coefficient was calculated by using the corresponding formula [56]:

(11) $$Synergistic\;coefficient=\frac{k(HC+H2O2)}{kHC\;+\;kH2O2}$$

**Table 3:** Results of MO degradation.

Operative conditions	Time of degradation [min]	Effective degradation rate constant, $k_{\mathrm{eff}}$ [min^−1^]	Per pass degradation factor Φ [-]	Degradation [%]	Synergistic coefficient [-]	$Y_{\mathrm{cav},HC}$[mg/kJ]
HC alone (1.5 bar)	60	0.0291	0.0890	83.3	–	3.65
MO/0.01 %v/v H_2_O_2_ alone	60	0.0001	0.0006	0.9	–	–
1.5 bar + 0.001 % v/v H_2_O_2_	60	0.0385	0.1091	89.4	1.32	3.93
1.5 bar + 0.005 % v/v H_2_O_2_	48	0.1051	0.1642	99.6	3.6	5.43
1.5 bar + 0.01 % v/v H_2_O_2_	36	0.1396	0.3279	99.8	4.78	6.77
1.5 bar + 0.1 % v/v H_2_O_2_	57	0.0759	0.2677	99.8	2.6	4.41
1.5 bar + 1 % v/v H_2_O_2_	60	0.0161	0.0487	63.6	0.55	2.68

There was a synergistic effect that occurred for the degradation of MO since the value of the effective rate constant of 0.0001 min^−1^ for the experiment utilizing MO/H_2_O_2_ alone, increased noticeably to 0.14 min^−1^ for the combination of hydrodynamic cavitation and H_2_O_2_. The synergetic coefficient for the optimum combination of HC/H_2_O_2_ (0.01 %v/v H_2_O_2_), was found to be 4.78, the highest in comparison with the other synergetic coefficients (Table 3). The hydrodynamic cavitation leads to the H_2_O_2_ dissociation, thus, the generation of more hydroxyl radicals, with a higher oxidation potential than radicals generated by H_2_O_2_ alone or HC alone.

The cavitation yield of HC alone at the pressure drop of 1.5 bar was found to be 3.65 mg/kJ, establishing a baseline for evaluating the impact of additional factors on the efficiency of the HC process. Combining HC with a low concentration of H_2_O_2_ (0.001 % v/v) results in an increased cavitation yield of 3.93 mg/kJ compared to HC alone, indicating a potential synergistic effect between HC and a minimal amount of H_2_O_2_. The optimum conditions seem to be at 0.01 % v/v H_2_O_2_, where the cavitation yield peaks at 6.77 mg/kJ, indicating an optimized synergistic effect between HC and H_2_O_2_ at this concentration. However, at a higher concentration (0.1 % and 1 % H_2_O_2_), the cavitation yield reduces to 4.4 mg/kJ and 2.67 mg/kJ respectively, suggesting that at these concentrations, H_2_O_2_ may exhibit a scavenger effect on MO degradation, impacting the overall cavitation yield, which is in accordance with the earlier explained interpretations. The results confirmed that the combination of hydrodynamic cavitation and H_2_O_2_, enhances the treatment performance of the MO degradation.

### 3.3 MO degradation by hydrodynamic cavitation, H_2_O_2_, and PC coupling

For exploring a possibility of further intensification of MO degradation, experiments were performed by combining optimal pressure drop across the vortex based hydrodynamic cavitation and optimum concentration of H_2_O_2_ with photocatalysis using TiO_2_ coated glass fibers. The TiO2-coated GFT, was activated by a UV source, enabling the formation of an electron/hole pair on its surface, which initiates the dissociation of water molecules through an electron transfer mechanism leading to a series of redox reactions, and consequently the production of more HO radicals in the system [57], in addition to those produced by HC/ H_2_O_2_, which increase the degradation of the organic pollutant present in the aqueous solution.

**Fig. 4:**
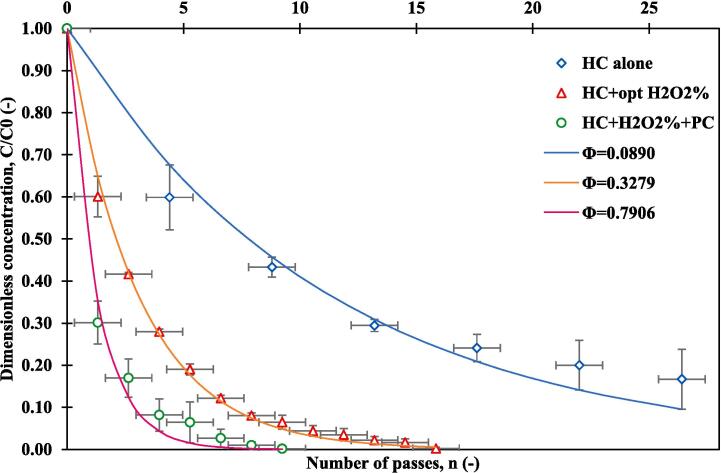
Degradation of MO using hydrodynamic cavitation alone, hydrodynamic cavitation + H_2_O_2_, and hydrodynamic cavitation + H_2_O_2_ + photocatalysis under agitation of 200 rpm using TiO2-coated GFT; V = 2.5 L, pH = 2 and MO initial concentration = 10 ppm, ΔP = 1.5 bar, and % H_2_O_2_ (0.01 %v/v).

The observed MO degradation with the three treatment methods namely, hydrodynamic cavitation alone, hydrodynamic cavitation combined with H_2_O_2_, and hydrodynamic cavitation, H_2_O_2_ coupled with photocatalysis, as a function of number of passes is shown in Fig. 4. For all these three systems, results are shown at corresponding optimum operating conditions: pressure drop (1.5 bar), H_2_O_2_ concentration (0.01 %v/v) and pH of 2. It can be seen from Fig. 4 that by using the hybrid system of HC/ H_2_O_2_ /PC, a degradation of 99.8 % was achieved after only 9 passes corresponding to 21 min, which is approximately three times faster than hydrodynamic cavitation alone, and almost 2 times faster when compared with HC/ H_2_O_2_, in terms of time.

The degradation rates, effective rate constant $k_{\mathrm{eff}}$, per pass degradation factor, treatment time, synergistic coefficients, and cavitation yield are listed in Table 4. It was observed that the effective rate constant of MO degradation with HC alone was 0.029 min^−1^, and 0.14 min^−1^ for HC/H_2_O_2_, whereas HC/ H_2_O_2_ /PC process was 0.27 min^−1^ corresponding to per pass degradation factors of 0.089, 0.328 and 0.79 respectively which confirm the generation of more OH**^.^** using the three AOPs together, twice as effective than the HC/ H_2_O_2_. The synergetic effect has been also observed between HC/H_2_O_2_ coupled with photocatalysis with a significant value of 9.2, this may be because HO**^.^** created by hydrodynamic cavitation and H_2_O_2_ contribute to the photocatalytic reaction. The synergistic coefficient realized under optimum conditions used in this work (9.2) is significantly higher than other reported values in the literature. For example, Fedorov et al. [58], reported the synergistic coefficient of 2.5 for the process of HC/H_2_O_2_/PC. The highest cavitation yield of 11.83 mg/kJ was observed when hydrodynamic cavitation, H_2_O_2_, and photocatalysis were combined. This indicates a further improvement in the degradation efficiency, emphasizing the synergistic effects of the hybrid system. The shorter time of degradation (21 min) supports the notion of enhanced pollutant removal efficiency in this combined approach, promising practical applications in wastewater treatment, particularly for textile industry effluents.

**Table 4:** MO degradation rates, effective rate constant $k_{\mathrm{eff}}$, per pass degradation factor Φ, and synergistic coefficients using the combination of HC, H_2_O_2_, and photocatalytic process in different treatment times.

Operative conditions	Time of degradation [min]	Apparent degradation rate constant, $k_{\mathrm{eff}}$ [min^−1^]	Per pass degradation factor Φ [-]	Degradation [%]	Synergistic coefficient [-]	$Y_{\mathrm{cav},HC}$[mg/kJ]
HC (1.5 bar)	60	0.0291	0.0890	83.3	–	3.65
HC/H2O2 (0.01 %v/v)	36	0.1396	0.3279	99.8	4.78	6.77
HC/H2O2/PC	21	0.2692	0.7906	99.8	9.22	11.83

### 3.4 Mineralization study

The results discussed so far were for degradation of MO calculated via monitoring the concentration of MO. It should however be noted that decrease in concentration of MO does not necessarily mean that complete mineralization has occurred. More often than not, complex molecules like MO will form several intermediates by reacting with oxidizing radicals before getting completely mineralized. It is therefore important to quantify the extent of mineralization in addition to MO degradation. Measurement of COD (chemical oxygen demand) is an extensively used analytical method for quantifying decomposition of organic carbon present in an aqueous solution. In this work, investigation of the quantitative mineralization was carried out under optimal conditions, for different systems such as HC alone, H_2_O_2_ alone, HC/ H_2_O_2_, HC/ H_2_O_2_ combined with a photocatalytic process. The discoloration of the MO solution during treatment results from oxidative attack on its chromophore (-N = N-), which breaks the two aromatic rings of the parent structure [59]. However, colorless does not mean complete mineralization has been achieved [60].

**Fig. 5:**
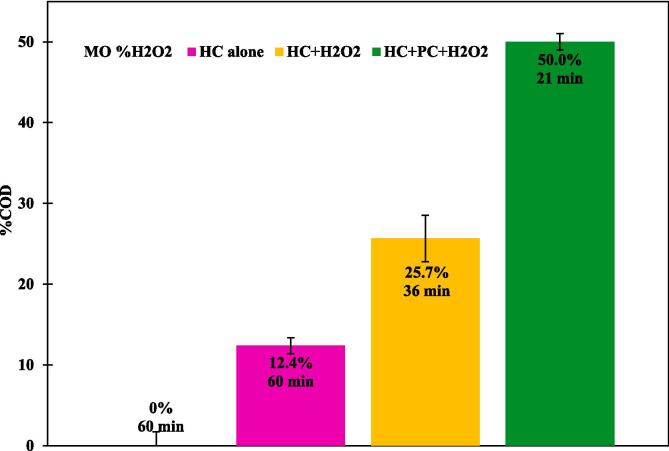
Effect of HC alone, H_2_O_2_ alone; HC/ H_2_O_2_, and HC/ H_2_O_2_ coupled photocatalysis on the degradation of COD in MO solutions.

Fig. 5 shows the obtained mineralization data in four diverse systems. it was observed that cod reduction rates of 0 % and 12.4 % were obtained for H_2_O_2_ alone and HC alone, respectively, over 26 passes corresponding to 60 min of treatment, and for HC/H_2_O_2_, the COD extent of mineralization increased, by more than factor two, to 25.7 % after 16 passes equivalent to 36 min. The maximum mineralization extent of 50 % was obtained for the HC/ H_2_O_2_/PC system, which is twice as high as that obtained for the HC/ H_2_O_2_ hybrid system and three times higher for the HC and H_2_O_2_ systems individually, within 9 passes corresponding to 21 min of treatment, and this confirms the obtained results in each series of experiments in our study. The increase in mineralization observed could be due to the generation of a greater number of OH radicals**.** The results presented here will be useful for designing a hybrid treatment process for desired mineralization of azo dyes.

It's important to note that the degradation experiments were conducted until achieving over 99.5 % degradation corresponded to approximately 50 % mineralization, after which accurate monitoring of dye concentration became not possible. Further treatment won't increase dye degradation since it's already fully degraded, but mineralization will continue to rise. Therefore, our methods can enhance mineralization, making the effluent suitable for discharge. The reported mineralization in this work surpasses that of other studies [61]. The results presented here will be useful for designing a hybrid treatment process for desired mineralization of azo dyes.

## 4 Conclusions

In this work, we investigated the degradation of MO dye solution using three AOPs at laboratory scale. The vortex-based cavitation device was used for hydrodynamic cavitation. The results obtained with HC alone were augmented by combining it with H_2_O_2_ and (H_2_O_2_ and PC). The key conclusions from this study are:

• MO degradation by HC (nominal capacity of 1 LPM) was found to be maximum when pressure drop across the device was 1.5 bar. Further increase in pressure drop reduced effective per-pass degradation factor. The pressure drop of 1.5 bar led to MO degradation of 96.4 %, at pH 2 with an extent of mineralization of 12.4 %.
• The addition of H_2_O_2_ to the MO solution, without hydrodynamic cavitation, showed only a weak degradation effect. Only 1 % of the MO was degraded with no apparent mineralization.
• The combination of hydrodynamic cavitation and H_2_O_2_ showed a significant synergistic effect of 4.78 at an optimum H_2_O_2_ concentration of 0.01 %v/v with a degradation yield of 99.8 %. The cavitation yield for HC alone was 3.65 mg/kJ, whereas the combined treatment of H_2_O_2_ + HC exhibited a higher cavitation yield of 6.77 mg/kJ. The extent of mineralization was increased to 25.7 %, after 16 passes corresponding to 36 min of treatment, higher than those achieved with HC and H_2_O_2_ separately.
• The hybrid system of HC/ H_2_O_2_/ PC, markedly enhanced the MO degradation with 50 % mineralization in 21 min equivalent to 9 passes, which is three times higher than using hydrodynamic cavitation alone and two times higher than using the combination HC/ H_2_O_2_, with a noticeable synergistic effect of 9.2 and important cavitation yield of 11.83
• The hybrid treatment comprising a combination of HC, H_2_O_2,_ and photocatalysis with TiO2-coated GFT was found to be most effective in degrading and mineralizing MO. Notably, the cavitation yield was significantly enhanced with this combined treatment.

Our work has shown that combining HC with different AOPs (H_2_O_2_, PC) is a promising solution, which not only accelerates MO decomposition but also improves cost-effectiveness due to the synergistic effect between the processes. The results obtained will be useful for researchers working on effluent treatment by coupling HC/ H_2_O_2_/PC.

## CRediT authorship contribution statement

**Ryma Merdoud:** Writing – original draft, Methodology, Investigation, Validation, Data curation. **Farid Aoudjit:** Supervision. **Lotfi Mouni:** Supervision. **Vivek V. Ranade:** Writing – review & editing, Supervision, Funding acquisition, Methodology, Conceptualization.

## Declaration of competing interest

The authors declare that they have no known competing financial interests or personal relationships that could have appeared to influence the work reported in this paper.
