# Supplementary material for: Degradation of methyl orange using hydrodynamic Cavitation, H2O2, and photo-catalysis with TiO2-Coated glass Fibers: Key operating parameters and synergistic effects
Source: Ultrason Sonochem. 2024 Jan 18;103:106772. doi: 10.1016/j.ultsonch.2024.106772 (PMC10847762; doi:10.1016/j.ultsonch.2024.106772)
Supplement: Supplementary data 1 [file mmc1.pdf]

Supplementary information

Degradation of Methyl Orange using Hydrodynamic Cavitation, H_2O_2 and Photo-catalysis: Key operating parameters and synergistic effects

Ryma Merdoud [a, b, c], Farid Aoudjit [a], Lotfi Mouni [b], and Vivek V. Ranade [c]*

[a] Laboratoire Matériaux et Développement Durable, Faculté des Sciences et Sciences Appliqués, Université de Bouira, 10 000 Bouira, Algeria
[b] Laboratoire de Gestion et Valorisation des Ressources Naturelles et Assurance Qualité, Faculté SNVST, Université de Bouira 10000, Algeria
[c] Department of Chemical Sciences and Bernal Institute, University of Limerick, Ireland

*Corresponding author: Vivek.Ranade@ul.ie

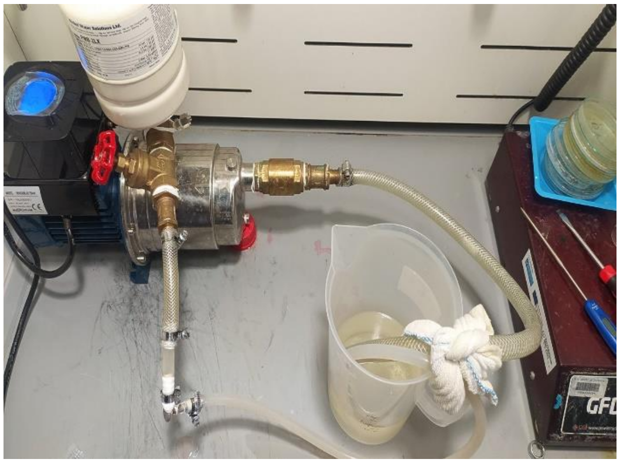

Fig.S1. Experimental setup of HC

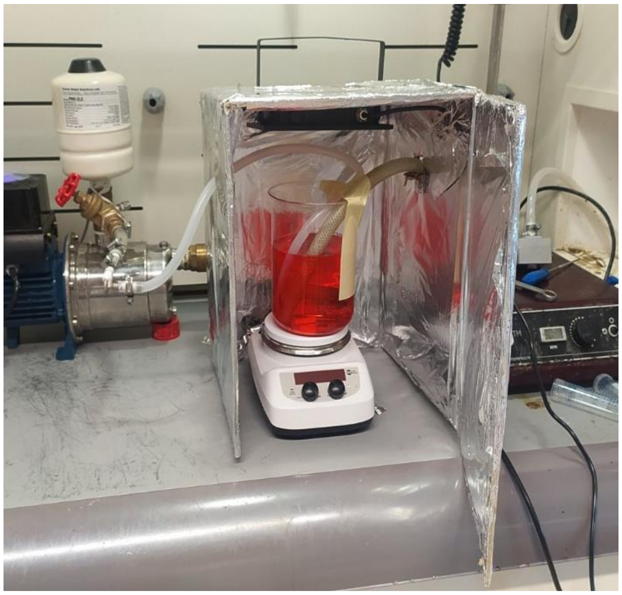

Fig.S2. Experimental setup of HC/PC

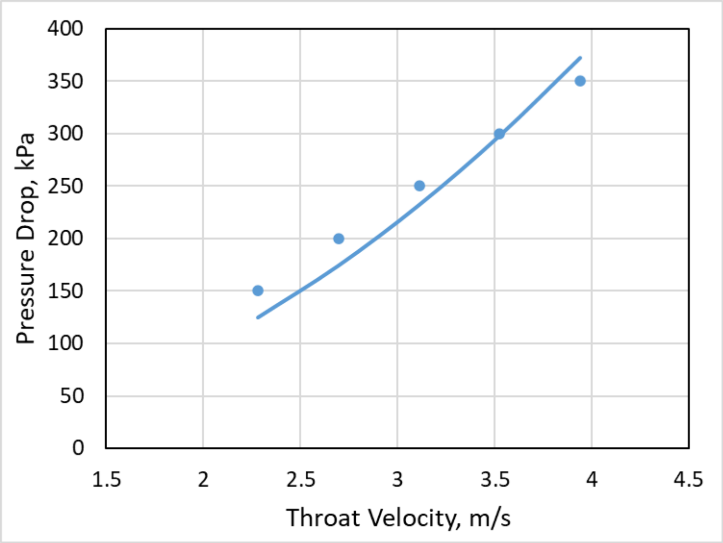

Fig.S3. Pressure drop and flow relationships for vortex based cavitation devices (Continuous line, Eu=48)

=

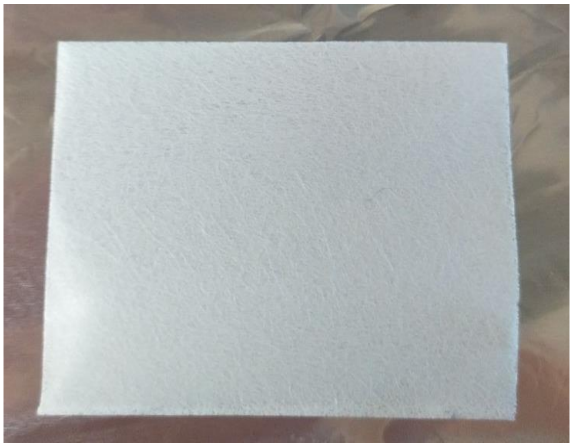

Fig. S4. TiO_2 coated GFT (25 cm^{-2})

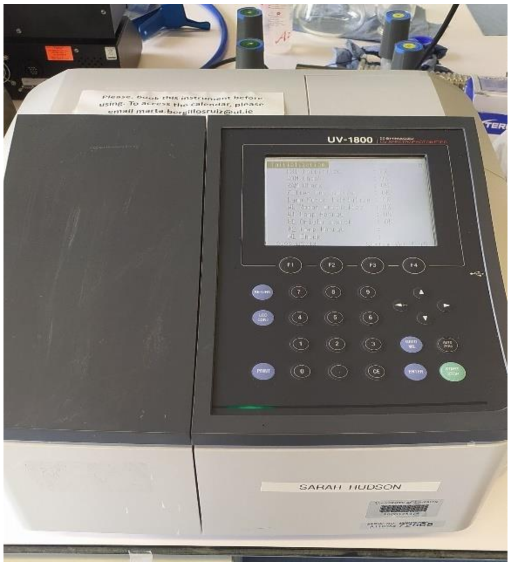

Fig. S5. UV spectrophotometer (Shimadzu UV1800)

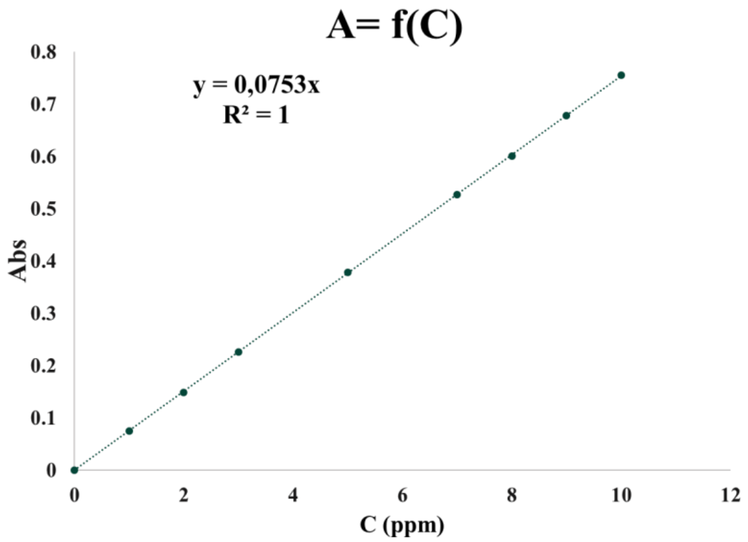

Fig. S6. MO calibration curve

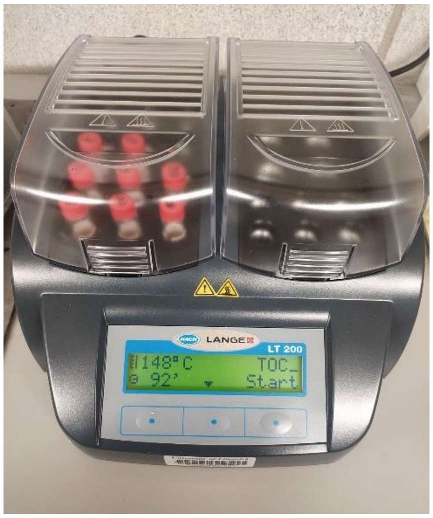

Fig. S7. DR 1900 spectrophotometer (Hach)

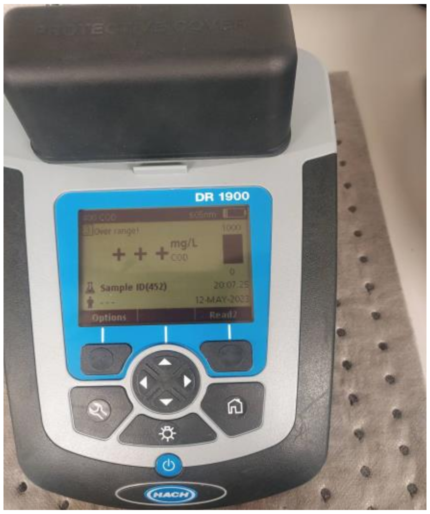

Fig. S8 LT 200 digester (Hach Lange, Germany)

S1. Characteristic of the photocatalyst

S1.1 XRD analysis

Fig.S9 illustrates the XRD diffraction pattern of GFT coated with a TiO_2 photocatalyst. The diffractogram obtained from TiO_2 on the GFT revealed the presence of intense peaks at 2θ = 25.42°, others less intense at 37.07°, 48.19°, and 54.85°, which are typical of the anatase form's crystalline structure of TiO_2, no rutile phase was found. **[1].** On the other hand, the diffractogram shows a broad peak due to the amorphous structure of the GFT. The average crystallite size of the TiO_2, is a vital parameter that influences photo-reactivity because it affects several physical properties of the nanoparticles such as surface area, surface energy, light absorptivity, and lattice distortion **[2].** Based on this characterization, the average crystallite size was estimated to be 6 nm using the Scherrer equation (**Eq. S1**) **[3],** which was found to be similar as reported by Yu et al. **[4]**. Interestingly, there are a lot of reports indicating that the optimum crystallite size for various photocatalytic reactions is in the range of 7–15 nm **[5].** However, it has been demonstrated that a reduced crystallite size leads to a higher surface area, which enhances the adsorption of reactants and, consequently, photo-reactivity **[6]**. Furthermore, it has been noted that the photogenerated radicals' energy increases as crystallite size is reduced due to the quantum confinement effect, which enhances photocatalyst performance **[7].**

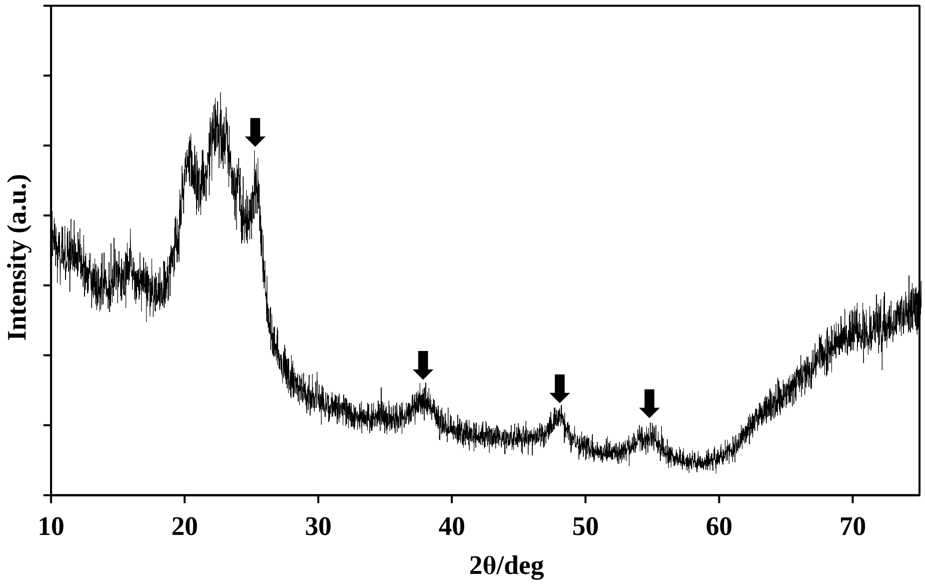

Fig.S9. XRD diffractogram of GFT coated with TiO2.

S1.2 Scanning electron microscopy

SEM is the best method for evaluating the homogeneity, degree of dispersion, degree of aggregation, and purity of nanoparticles. **[8,9]**. **Fig.S10** shows SEM images of the surface morphology of the photocatalyst. It clearly depicts the support's fibrous structure (glass fibers) as well as the heterogeneous dispersion of the TiO2 nanoparticles, with different sizes, as thin films, on its surface (**Fig. 2a and 2b**) as reported by Satoru et al. **[10]**. By zooming in on the fiber surface, the TiO2 clusters are visible (**Fig. 2c**), which were used in the photocatalysis process to degrade MO present in synthetic solution.

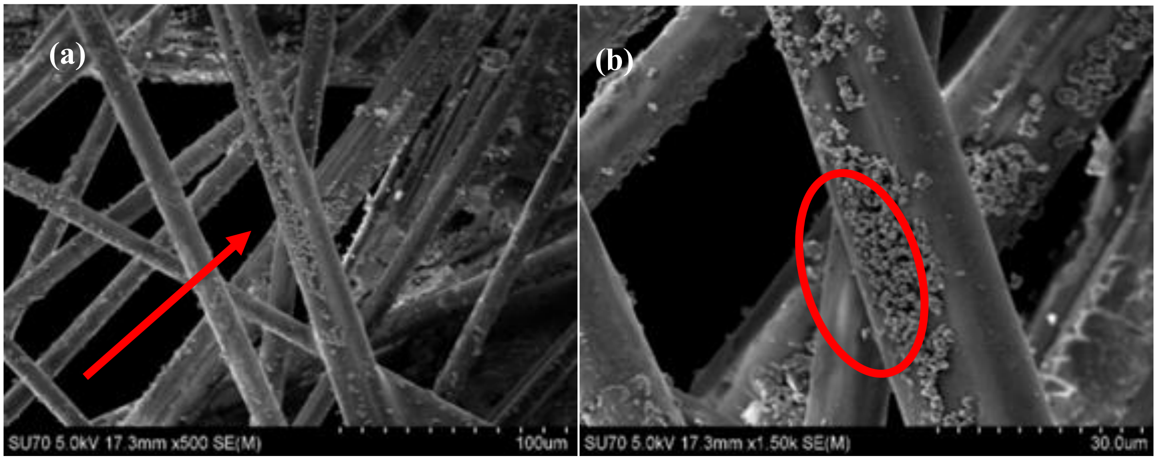

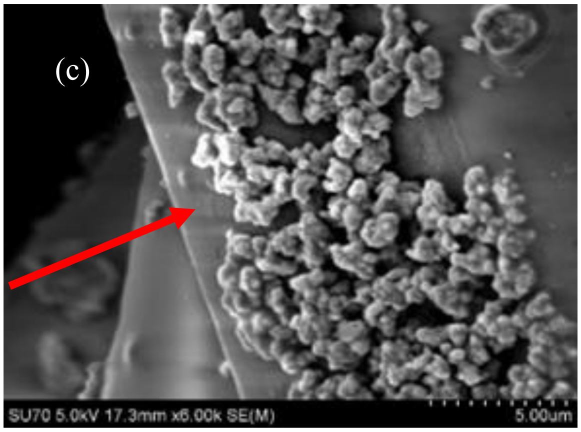

Fig.S10. SEM images of TiO2 coated on GFT×500 (a) ×1.5K (b) ×6.00K (c)

- **The crystallite size calculated using the Scherrer equation:**

$$D = \frac{0.9\lambda}{(\beta\cos\theta)} \quad \textbf{(S1)}$$

Where D (nm) is crystallite size, K is Scherrer constant, λ (0.15406 nm) is the the wavelength of the X-ray sources, β (radians) is the Full width at half maximum (FWHM), and θ (radians) is the peak position.

θ (radians)	β (FWHM)	D (nm)	D average (nm)
25.29264	1.58753	5.13	5.97
37.91085	2.96098	2.84	-
48.07368	1.88648	4.61	-
54.16614	0.78976	11.3	-

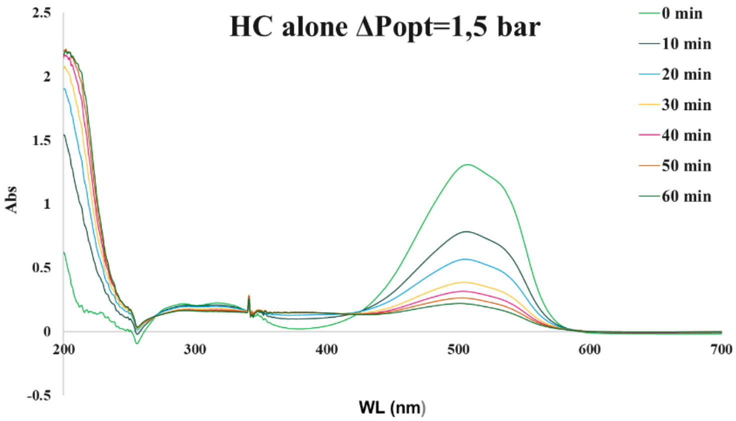

Fig. S11. MO UV spectrum at optimum pressure drop of 1.5 bar; pH=2, and initial concentration = 10 ppm

References

[1] N . Belkessa, Y . Serhane , A . Bouzaza , L . Khezami , A . A . Assadi, Gaseous ethylbenzene removal by photocatalytic TiO2 nanoparticles immobilized on glass fiber tissue under real conditions: evaluation of reactive oxygen species contribution to the photocatalytic process, Environ Sci Pollut Res Int. 30 (13) (2023 Mar) 3574 5 –3575 6 https://doi.org/10.1007/s11356 - 02 2 -2463 6 - 8 , Epub 2022 Dec 20 PMID : 36538222 .
[2] M . A . Henderson , A surface science perspective on TiO2 photocatalysis, Surf. Sci. Rep. 66 (6 – 7) (2011) 18 5 –29 7.
[3] M . Music , M . Gotic , M . Ivanda, S . Popovi c , A . Turkovic , R . Trojko , A . Sekuli c , K . Furi c , Mat. Sci. Eng. b: Soli d 47 (1997) 33 .
[4] H . Yu , S . Le e , J . Yu , C . Ao , Photocatalytic activity of disperse d TiO2 particle s deposite d on glas s fibers , J. Mol. Catal. A Chem . 24 6 (1 – 2) (2006) 20 6 –21 1 , https://doi.org/10.1016/j.molcata.2005.11.00 7 .
[5] S . Li u , N . Jaffrezi c , C . Guillard , Size effect s in liquid -phas e phot o -oxidatio n of phenol usin g nanomete r -size d TiO2 catalyst s , Appl . Surf. Sci. 25 5 (5) (2008) 2704 –2709 , https://doi.org/10.1016/j.apsusc.2008.07.191 .
[6] M . Xi e , L . Jing , J . Zhou , J . Li n , H . Fu , Synthesi s of nanocrystallin e anatas e TiO2 by on e -po t tw o -phas e separate d hydrolysis -solvothermal processe s an d it s high activity fo r photocatalytic degradatio n of rhodamin e B , J. Hazard . Mater. 17 6 (1 – 3) (2010) 13 9 –14 5 , https://doi.org/10.1016/j.jhazmat.2009.11.00 8 .
[7] J . Ji u , F . Wang , M . Adachi , Preparatio n of highly photocatalytic active nano - scal e TiO2 by mixe d template method , Mater. Lett . 58 (30) (2004) 3915 –3919 , https://doi.org/10.1016/j.matlet.2004.08.017 .
[8] V . Patraval e , P . Dandekar , R . Jain , Characterization techniques fo r nanoparticulat e carriers , Nanoparticulat e Drug Delivery 87 –12 1 (2011) , https:// doi.org/10.1533/9781908818195.87 .
[9] A . E . Vladár , V . Hodoroab a , Characterization of nanoparticle s by scanning electron microscopy , Characterization of Nanoparticle s 7 –27 (2019) , https:// doi.org/10.1016/B978 - 0 -12 -814182 -3.0000 2 - X .
[10] S . Fukugaichi , Fixation of titanium dioxid e nanoparticle s on glas s fibe r cloths fo r photocatalytic degradatio n of organi c dyes , AC S Omeg a 4 (2019) 1517 5 –1518 0 .
